# Corrosion Features of the Reinforcing Bar in Concrete with Intelligent OH^−^ Regulation of Microcapsules

**DOI:** 10.3390/ma12233966

**Published:** 2019-11-29

**Authors:** Shuxian Hong, Shaofeng Qin, Biqin Dong, Feng Xing

**Affiliations:** School of Civil Engineering, Shenzhen University, Shenzhen 518060, China; sxhong@szu.edu.cn (S.H.); dongbq@gmail.com (B.D.)

**Keywords:** reinforcement, concrete, corrosion, microcapsule, X-ray computed tomography (XCT)

## Abstract

Corrosion is a challenging problem for marine concrete infrastructure projects. In this study, an intelligent OH^−^-regulated microcapsule is designed to prevent reinforcement corrosion, taking ethylcellulose (EC) as shell material and calcium oxide (CaO) as core material. X-ray computed tomography (XCT) is used to trace and contrast the corrosion profiles of the concrete reinforcement bar with and without the microcapsule. The results show that the OH^−^-regulated microcapsule exhibits effective corrosion protection by delaying corrosion initiation and cracking. An SEM study revealed that the microcapsule could be broken as Cl^−^ invades the concrete. However, intelligent OH^−^ regulation was realized by releasing CaO.

## 1. Introduction

Reinforced concrete has been widely applied in civil engineering projects due to its complementary advantages [1]. However, reinforcement corrosion has become a major threat to the durability of reinforced concrete, particularly marine splash zones, where it is subjected to cyclic wetting and drying with chloride permeation [1,2]. Normally, in the highly alkaline environment of the concrete, the reinforcement bar is protected from corrosion by a thermodynamically stable film. Nevertheless, a high corrosion risk may occur when the alkalinity is locally compromised, such as when chloride permeation or carbonation occurs [3,4]. Such processes will shorten the service life of concrete infrastructure, arousing the attention of researchers in construction engineering [1,5,6,7].

Traditionally, corrosion inhibitors, such as nitrites or sodium monofluorophosphate, have been frequently used to improve resistance to chloride invasion [8,9,10,11]. The addition of inhibitors to the concrete could strengthen the passive film on the reinforcing bar, and reduce oxygen and chloride invasion on the surface due to the oxidization of nitrite ions [8,9] or the precipitation of ferric phosphate (FePO4) [8,11]. This would delay the corrosion process. However, some drawbacks exist due to difficulties in the direct incorporation of these corrosion inhibitors. These inhibitors can be less durable due to the diffused outflow of nitrite ions [12], or they can participate in reactions during the hydration process of the cement, which could adversely impact the microstructure of the hydration products [13,14]. To address this problem, Dong proposed and designed a protective barrier for corrosion inhibitors using an enmicrocapsulation technique that avoids unexpected depletion and reaction during early hydration [15,16].

Additionally, numerous researchers have pointed out that the initiation of corrosion in reinforced concrete is primarily affected by the threshold value of the Cl^−^/OH^−^ ratio [17,18,19,20,21,22,23,24,25,26], indicating that corrosion initiation could be repressed by increasing the OH^−^ concentration in concrete materials. The fundamental theories regarding this hypothesis consist of the following: firstly, a dynamic equilibrium exists between the breakdown of the passive film due to chloride ingression and the maintenance of the passive status of the reinforcing bar because of the high content level of OH^−^. In addition, the breakdown of the passive film initiates when an increased amount of Cl^−^ reaches and simultaneously leaches OH^−^ from the surface of the reinforcing bar [17]. Secondly, C_3_A is considered one of the main ingredients affecting the chloride binding capacity of cement, but a decrease in the alkaline content would compromise the chloride binding capacity of C_3_A, causing an increase in free chlorides [18,23]. And thirdly, chloride-induced corrosion is an electrochemical process (the breakdown of passive film is regarded as the active zone, while the surrounding passive film is considered the cathodic zone), and the increase in OH^−^ content could inhibit the cathodic reaction, thereby decreasing the corrosion rate [8,11,13,14]. From this aspect, OH^−^ can be regarded as a corrosion inhibitor [8,11]. Whereas, OH^−^ could not be supplied without limit because of the decrease in OH^−^ content and calcium leaching under wet-dry cycles in the splash zone [27], though calcium hydroxide is a hydration product of cement that acts as a pH buffer. Furthermore, the hydrolysis of free ferrous ions causes local acidity in the active zone, aggravating the decrease of the OH^−^ content in the concrete due to acid–base neutralization.

Although numerous papers have mentioned corrosion inhibition due to high alkalinity [8,11,13,14], fewer investigations have discussed the supplementation of OH^−^ as a corrosion inhibitor in concrete. 

**Figure 1:**
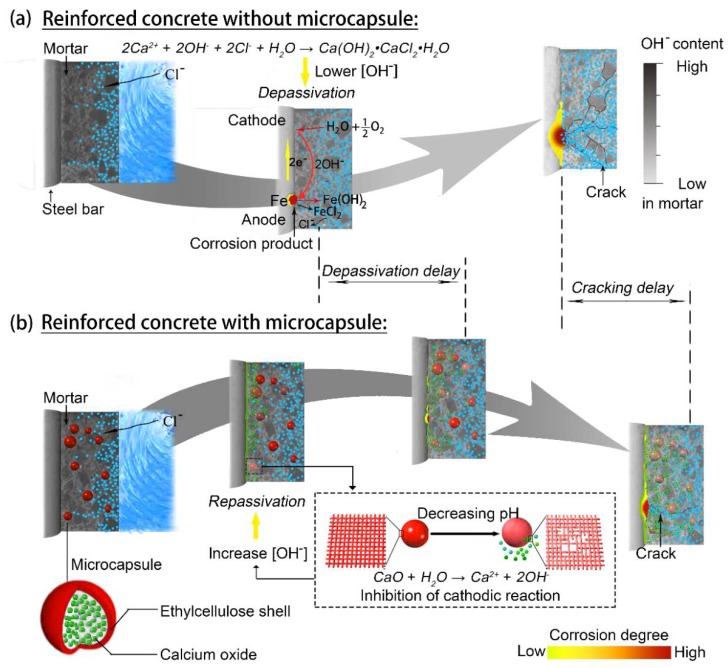
The schematic of OH^−^-regulated intelligent microcapsules for the corrosion protection of the reinforcing bar in reinforced concrete: (**a**) without microcapsules and (**b**) with microcapsules.

In this study, a new style of OH^−^ regulation, in combination with enmicrocapsulation, is proposed to achieve high-efficient corrosion protection for the reinforcement bar in concrete. The functional schematic is presented in Figure 1b. For comparison, chloride-induced corrosion in a non-functionalized matrix is also illustrated in Figure 1a. For this kind of microcapsule, a pH-sensitive ethylcellulose (EC) was selected for the shell material, which will keep stable at high pH levels, but break down with a decrease in the pH value [28,29]. Calcium oxide (CaO) was used as the core material, which will be released when the shell material breaks down. Hydroxyl ions will be generated due to the following chemical reaction, see Equation (1):

CaO + H_2_O→Ca^2+^ + 2OH^−^ (1)

The EC/CaO microcapsule satisfies the requirements of chloride-induced corrosion in concrete materials and is able to regulate OH^−^ in concrete materials. In order to simulate coastal conditions, in particular the splash zone, cyclic wet-dry tests were performed. Previous researchers have verified that EC-film microcapsules encapsulating traditional corrosion inhibitors could be triggered and release core agents under the wet-day cycles [15,16]. In addition, X-ray computed tomography (XCT) was used to periodically trace the samples and contrast the corrosion profiles of the concrete reinforcement bar with and without microcapsules. The efficiency of corrosion protection was then quantitatively analyzed [30].

## 2. Materials and Methods 

### 2.1. Microcapsule Fabrication

The core and shell materials of the EC/CaO microcapsule are shown in Table 1 and Table 2, respectively. The microcapsules were primarily manufactured using the cold extrusion-spheronization and spray-drying film-forming technique.

CaO, microcrystalline cellulose (MCC), hydroxypropyl methylcellulose (HPMC), and polysorbate 80 were prepared to be used as the core materials with a ratio of 57:39:2:2. The materials were dissolved in 60% (w/v) ethanol at a ratio of 2:3. After uniform mixing, the core material was then formed into the core particles using the cold extrusion-spheronization method with high-speed centrifugation. Microcrystalline cellulose was used as the framework for the microcapsule core, while hydroxypropyl methylcellulose and polysorbate 80 were used as binders.

Ethanol and methylbenzene were mixed at a ratio of 1:4 to be used as the EC solution [31]. The solvent of the shell materials was evaporated during the spray-drying process so that the EC remained on the particle surface of the microcapsule cores, thus forming the microcapsule shells. Bits of talcum powder were incorporated to prevent adhesion between the microcapsules. The EC/CaO microcapsules were successfully fabricated after natural drying.

A laser particle size analyzer (BaiTe Instruments, BT-9300ST, Liaoning, China) was used to investigate the size distribution of the EC/CaO microcapsules. The morphology of the microcapsules was observed using an optical microscope (CAIKON, XTL-3000C, Shanghai, China) after mixing with mortar.

### 2.2. Specimen Preparation

**Figure 2:**
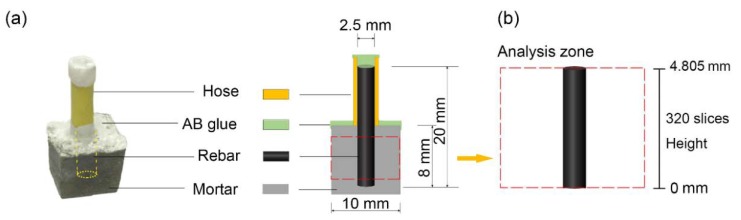
Basic information of the sample: (**a**) photograph of the sealed sample and the profile schematic and (**b**) the selected analysis zone for further X-ray computed tomography (XCT) analysis.

Two types of samples were prepared for testing, one control sample without microcapsules (Sample A) and three testing samples with different percentages of CaO/EC microcapsules (0.5 wt%, 3 wt%, and 5 wt% of the cement corresponding with Samples B, C, and D, respectively), considering not only effects of the microparticles on the porosity, as well as mechanical properties of samples, but also the possible anti-corrosion efficiency of the samples [32]. Additionally, the water/cement/sand (W/C/S) ratio in all the samples was 2:5:5, as listed in Table 3. Figure 2a shows the size of a sample, which was 10 mm × 10 mm × 10 mm. A 2.5 mm diameter reinforcing bar was embedded 8 mm deep in the sample. Figure 2b shows the analysis zone of each reinforcing bar used for the investigations. In this study, a Q235 reinforcing bar was used that follows the Chinese standard GB/T3274-2007, with the elemental compositions listed in Table 4. The chemical composition of the cement is given in Table 5. After 24 h curing in a curing chamber (95 ± 5% RH, 20 ± 2 °C), these samples were de-molded, sealed with a hose and AB glue (A: acrylate monomers; B: curing agents), and then cured for 28 days under the same conditions.

### 2.3. Accelerated Corrosion

A wet-dry cyclic test was performed to accelerate corrosion of the samples. The samples were immersed in a 3.5 wt% NaCl solution for three hours, and then placed in an electric oven with a relative humidity of 50% at 50 °C and dried for another three hours. An accelerated corrosion test with 3 h wet and 3 h dry cycles was repeated, and an entire wet-dry cycle contained 12 total wet-dry cycles (72 h).

### 2.4. XCT Testing

The XCT technique was used to periodically trace the accelerated corrosion process. The initial state of the samples was monitored using XCT measurements before the first wet-dry cyclic test. After each 72 h wet-dry cycle, XCT testing was carried out. The experiment (wet-dry cyclic acceleration and the XCT method) was repeated until external cracks appeared.

**Figure 3:**
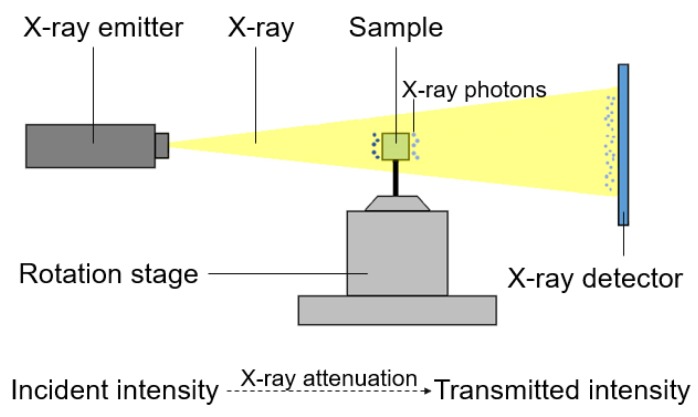
An illustration of the internal setup of the XCT imaging system.

The internal instruments of the XCT (Micro XCT-400; XRadia Inc, CA, USA) primarily consist of a microfocus X-ray emitter, a 360-degree imaging rotation stage, and an image intensifier detector with a charge-coupled device (CCD) camera, and these comprise an image processing unit. XCT measurement is illustrated in Figure 3, and the relative principles of XCT measurements have been illustrated in previous reports [30,33,34,35]. The X-ray attenuation power in each voxel is expressed as the gray value in the XCT image, which represents the X-ray photon absorption degree of the material. Basic information regarding XCT measurements is listed in Table 6.

### 2.5. Linear Polarization Testing

After the initial 3 h wetting of each 72 h wet-dry cycles, the linear polarizations of three parallel samples without and with the microcapules were tested at a steady open circuit potential (OCP) by using an electrochemical workstation (AMETEK, Parstat, MC, USA). Polarization measurements tests were executed to investigate the anti-corrosion properties in the potential range of ±20 mV versus OCP with a 0.1666-mV·s^−1^ potential scanning rate. The polarization data in the potential range of ±10 mV versus OCP was fitted by using Cview software, and linear polarization resistance (*R*_p_) was then obtained. In addition, the corrosion current density (*i*_corr_) could be gained through the following Equation (2):

(2) $$i_{corr}=B/R_{p}$$

where *B* is a constant 26 mV for the bare steel in active condition and 52 mV for bare steel in the passive state [36]. It should be noted that polarization measurements would stop after the cracking of all the control samples occurred and the cracks expanded outside.

### 2.6. ESEM Testing of the Microcapsules and the Corroded Samples

An environmental scanning electron microscopy (ESEM; Quanta FEG 250, FEI, Hillsboro, OR, USA) system was used to observe the morphology of the EC/CaO microcapsules before and after hydration. An equipped texture element analysis microscopy (TEAM) apparatus (EDAX, Mahwah, NJ, USA) was used to characterize the chemical composition of the sample. After the final XCT testing was completed, the samples were sliced and examined using ESEM and TEAM.

## 3. Results

### 3.1. Basic Investigation of the Microcapsules

**Figure 4:**
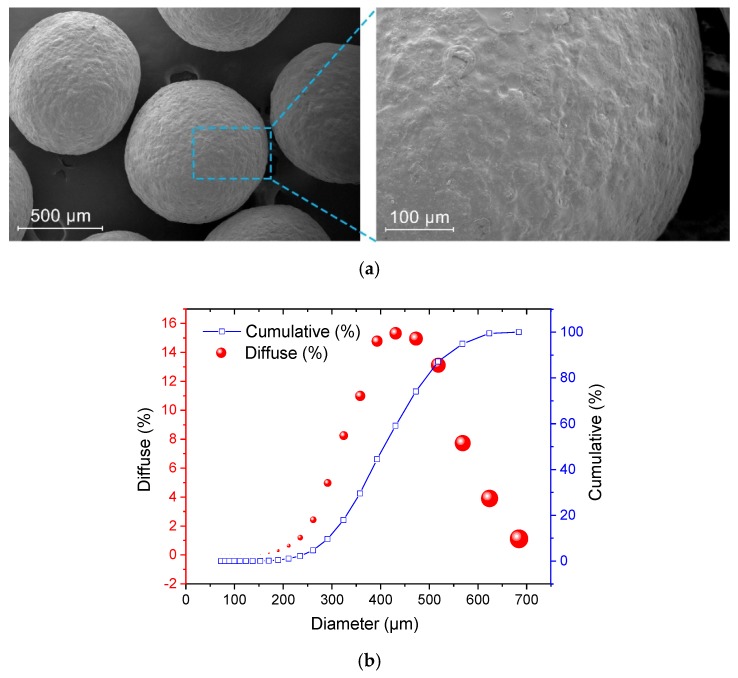
Basic investigations of the CaO/ethylcellulose (EC) microcapsules: (**a**) morphology of the microcapsule obtained from environmental scanning electron microscopy (ESEM) measurements and (**b**) particle size distribution of the microcapsules.

**Figure 5:**
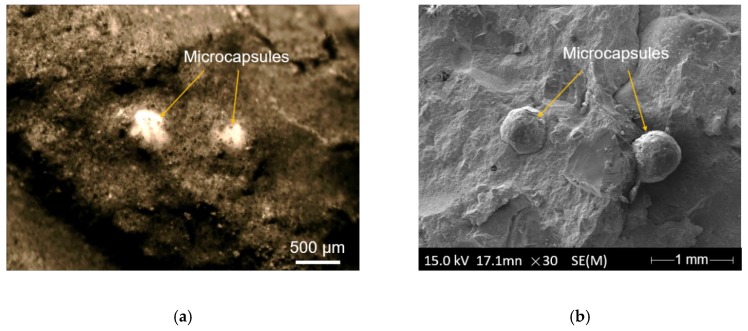
Morphology of the CaO/EC microcapsules: (**a**) optical microscope observation after mixing with the mortar and (**b**) fracture surface of the mortar matrix found using the ESEM measurements after cement hydration and hardening for 28 days.

The morphology of a CaO/EC microcapsule is presented in Figure 4a. The microcapsules had a rough surface that enhanced the bond between the microcapsule and mortar. Figure 4b shows that the sizes of the microcapsules primarily were in the range of 400–600 µm, with a mean diameter of 407 µm [37]. Figure 5a shows that the microcapsules retained their spherical shape after they were mixed with the mortar. The microcapsules maintained an intact morphology and a desirable bonding ability with the mortar matrix. Figure 5b shows that two microcapsules adhered to the mortar where they maintained their spherical shape after 28 days of hydrating and hardening of the mortar.

### 3.2. Evaluation of the Efficiency of the Intelligent OH^−^-Regulated Microcapsules

**Figure 6:**
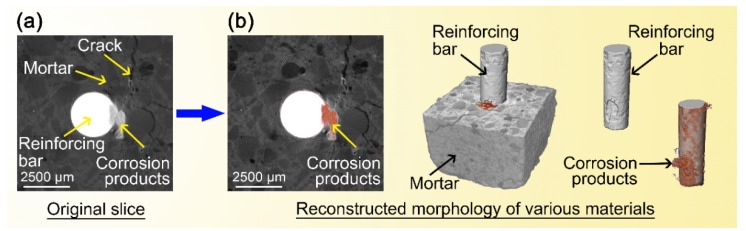
Images obtained from the XCT measurements and material characterization: (**a**) original slice and (**b**) reconstructed morphology to distinguish different substances (mortar, reinforcing bar with and without corrosion products).

The corrosion morphology of the sample obtained using the XCT measurement is shown in Figure 6. The original slice, shown in Figure 6a, was processed with image segmentation to distinguish different materials, including the reinforcing bar, corrosion products, the cement matrix, and cracks (Figure 6b). Thus, two-dimensional (2D) and three-dimensional (3D) information (the corrosion morphology of the reinforcing bar, development of corrosion products, and information on cracks) in the different samples could be vividly characterized.

**Figure 7:**
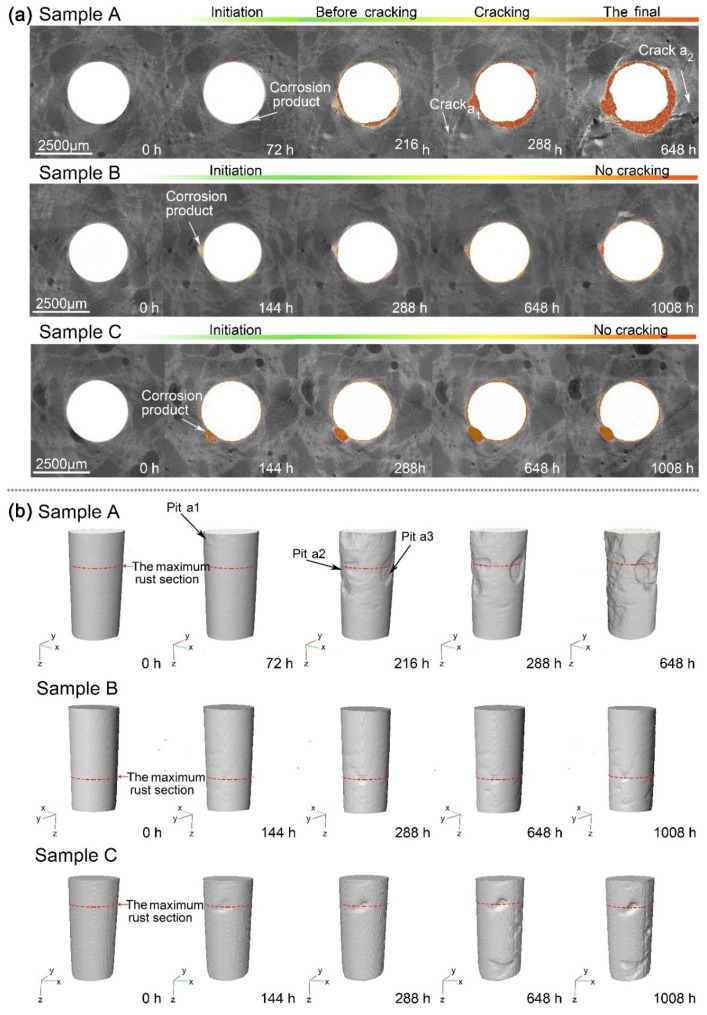
XCT results of different samples: (**a**) 2D images of the maximum corroded section and (**b**) 3D pattern of the corresponding reinforcing bars (the red dash line represents the corresponding maximum corroded section in Figure 7a).

Based on the XCT images, the corrosion process is visualized in Figure 7. The 2D morphological changes of the maximum corroded section in the control sample (Sample A) are exhibited in Figure 7a. Therefore, the corrosion initiation and cracking time can be determined. Prior to the first wet-dry cycle test (the initial state), the overall surface of the reinforcing bar was smooth and without rust. After 72 h of the wet-dry cycles, corrosion initiated on the surface of the reinforcing bar, and corrosion products appeared around the bar. After 288 h of continuous cycles of the wet-dry test, the expansive stress finally resulted in the cracking of the mortar matrix due to the accumulation of the corrosion product. A small amount of the corrosion products penetrated into the cracks (crack a1) in the mortar matrix. After 648 h of accelerated corrosion, as the cracks (cracks a1 and a2) developed, the corrosion process accelerated. The corrosion process is depicted in Figure 7b in corresponding 3D images.

In the samples with the microcapsule (Samples B and C), the corrosion initiation time was delayed to 144 h (Figure 7a), which is 72 h later than that of the control sample. After 1,008 h wet-dry cycles, the corrosion process of Samples B and C developed much more slowly than that of Sample A, and no obvious cracks were observed in the 2D images of Samples B and C. The corresponding 3D corrosion morphology of the reinforcing bar is also exhibited in Figure 7b.

To further access the protection efficacy of the intelligent OH^−^-regulated microcapsule, the XCT images were quantitatively analyzed using a method that has been verified using the gravimetric method derived in previous research [29,33]. The area of the reinforcing bar in different slices is represented as Equation (3):

(3) $$A_{i}=\;n_{A}\;\times \;Z_{A}$$

where *A_i_* is the area of the reinforcing bar in different slices (*i* represents the different XCT testing times, *i* = 0 h, 72 h, 144 h, and so on), *n*_A_ is the number of the pixels of the reinforcing bar in each slice, and *Z*_A_ represents the real area of one voxel (15.0173 × 15.0173 μm^2^).

**Figure 8:**
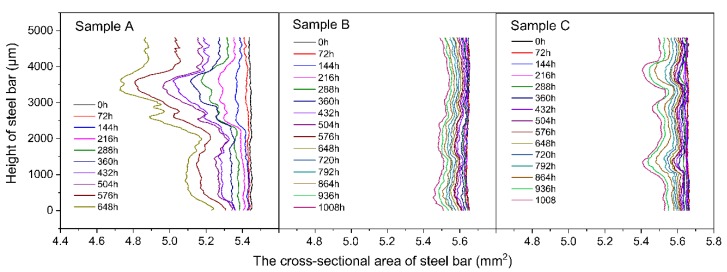
Cross-sectional area of the reinforcing bar along the height at different testing times.

The changes in the cross-sectional area, *Ai*, of the reinforcing bar along the height of the steel bar at different testing times are shown in Figure 8.

The area loss of the corroded reinforcing bar can be calculated as Equation (4):

(4) $$∆A=A_{0}-\;A_{i}$$

where ∆*A* is the area loss of the corroded reinforcing bar in each slice, *A*_0_ is the area of the reinforcing bar in each slice at 0 h (the cross-sectional area of the reinforcing bar at 0 h in Figure 8), and *A_i_* is the area of the reinforcing bar of each slice at different XCT testing times during the acceleration test.

**Figure 9:**
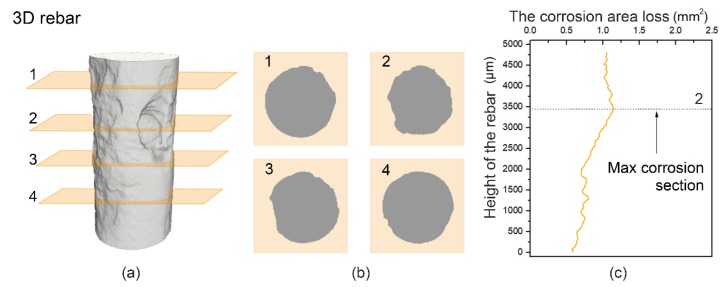
Morphology of the reinforcing bar of Sample A at 648 h and the quantitative analysis: (**a**) 3D morphology, (**b**) 2D morphology of the reinforcing bar from the four slices from Figure 9a, and (**c**) determination of the maximum corrosion section based on corrosion area loss.

The area loss of the reinforcing bar for each section was calculated through Equations (3) and (4). The maximum corroded cross-section for each sample was then obtained, and these are shown in Figure 9. With the progress of wet-dry cycles, these curves fluctuated in the height direction. This means that some pitting corrosion occurred and expanded during the corrosion process. The maximum area loss of the reinforcing bar for Sample A was approximately 0.7 mm^2^ at 648 h, as shown in Figure 8A. In a range of heights from 2500–4000 µm, dramatic loss occurred in the cross-sectional area due to corrosion. In Samples B and C at 1008 h, the cross-sectional area loss of the maximum corroded section was only 0.20 mm^2^ and 0.25 mm^2^, respectively (Figure 8B,C). During the entire experiment, in the control sample, the corrosion developed more locally. While in the samples with microcapsules, the corrosion occurred relatively uniformly along the height of the reinforcing bars. These results were in agreement with the 3D visualized results in Figure 7.

The total areas of the reinforcing bar were then multiplied by the voxel height (15.0173 µm) to obtain the volume of the reinforcing bar, *V_i_*. The volumetric loss of the corroded reinforcing bar, ∆*V*, was obtained using the following Equation (5):

(5) $$∆V=V_{0}-\;V_{i}$$

where *V*_0_ is the volume of the reinforcing bar at 0 h and *V_i_* is the volume of the reinforcing bar at different times during the acceleration test.

**Figure 10:**
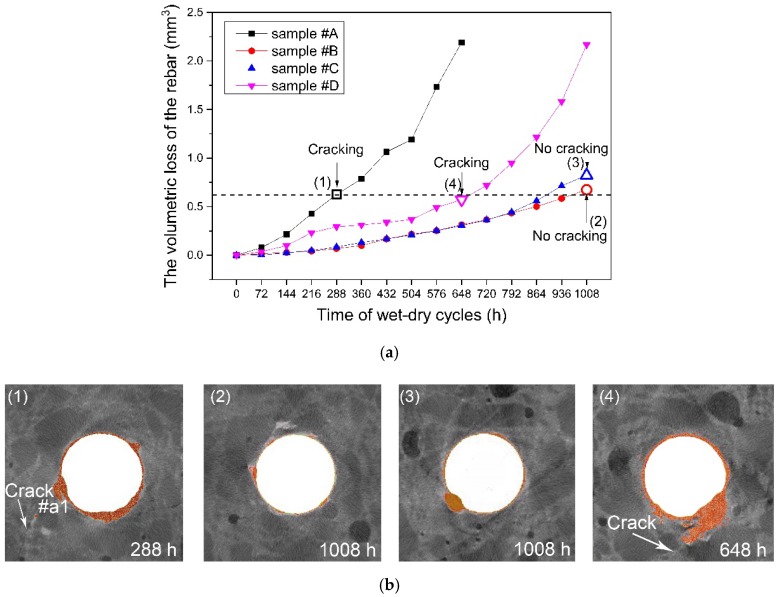
A quantitative analysis of the time-dependent corrosion of the four samples: (**a**) volumetric loss of the reinforcing bar with different wet-dry cyclic times and (**b**) maximum corroded section of different samples corresponding to the points in Figure 10a (with a similar volumetric loss around 0.626 mm^3^).

The obtained volume and volumetric loss of the reinforcing bar for the samples are listed in Table 7. The time-dependent volumetric loss of the corroded reinforcing bar is plotted in Figure 10, which shows that the corrosion process in the samples with microcapsules developed much slower than that in the control sample. These results correspond with the results shown in Figure 7 and Figure 8. In Samples B and C, no crack was observed during the entire 1008 h of the accelerated corrosion experiment, while Sample A reached the same volumetric loss at 288 h and cracks were obvious (Figure 10b).

**Figure 11:**
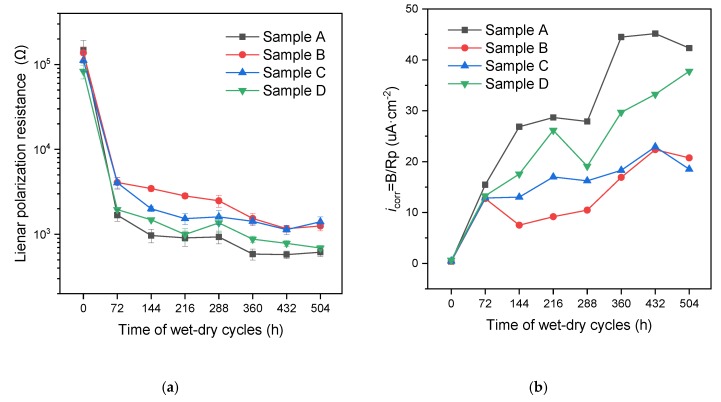
(**a**) Linear polarization resistance (*R*_p_) and (**b**) corrosion current density (*i*_corr_) of the four kinds of samples over wet-dry cyclic times.

Additionally, Figure 11 presents the *R*_p_ and *i*_corr_ of different kinds of samples through the wet-dry cycles. From the linear polarization results, all samples with OH^−^-regulated microcapsules showed higher linear polarization resistance (*R*_p_) than that of the control samples. The *R*_p_ of samples fluctuated during a specific period, mainly due to the OH^−^ regulation, which contributed to a decrease in the [Cl^−^]/[OH^−^] value and then alleviated the corrosion development. In contrast, without the help of OH^−^-regulated microcapsules, corrosion initiation and propagation of the control samples occurred more easily. The adverse trend of *i*_corr_ was shown in Figure 11b. These electrochemical results were basically in accordance with the results from the XCT measurements.

## 4. Discussion

**Figure 12:**
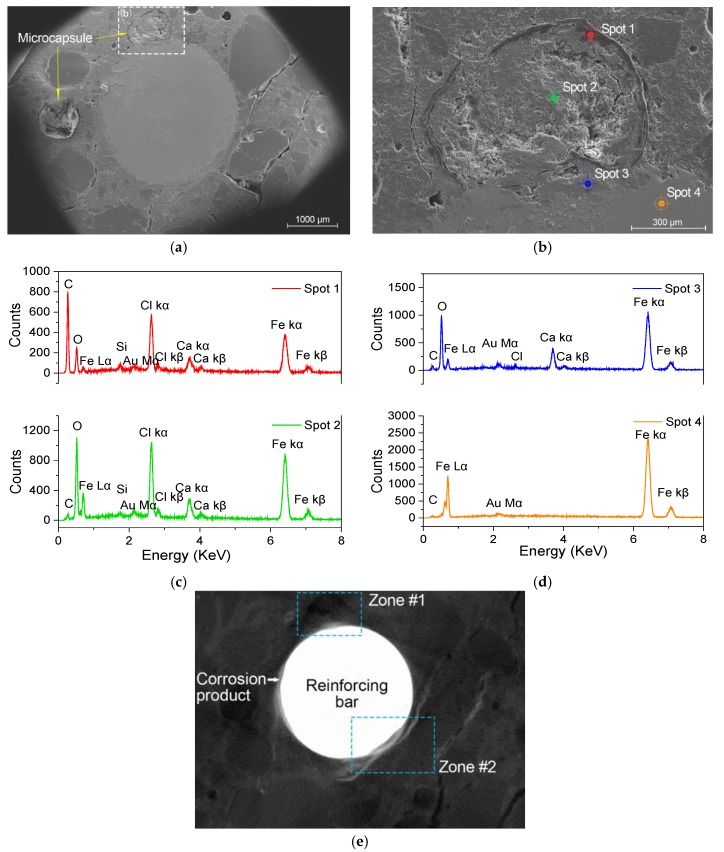
ESEM results with energy-dispersive X-ray spectroscopy (EDS) analysis after the entire accelerated corrosion procedure: (**a**) ESEM images of the cross-section of Sample D; (**b**) enlarged region of Figure 12a; (**c**,**d**) EDS analysis of the surface obtained using texture element analysis microscopy (TEAM) with spots 1, 2, 3, and 4 corresponding to the region of the capsule shell, OH^−^-regulated core, corrosion products, and reinforcing bar, respectively; and (**e**) the corresponding XCT scanning images.

During cement hydration, the pore solution was highly alkalized (pH > 12.5) and was principally comprised of sodium and potassium hydroxides [1,3]. It has been shown that the EC shell can resist high pH. Thus, the CaO/EC microcapsules are very stable in a highly alkaline environment [28,29], and they maintain their spherical shapes during mixing and hydration of the mortar (Figure 5). The SEM image shows that, after the wet-dry cycles, EC shells are damaged (Figure 12a,b), and Cl^−^ penetrated through the mortar matrix, as Cl element was found in the energy-dispersive X-ray spectroscopy (EDS) results (Figure 12c,d). Thus, CaO was released and reacted with water. The reaction product, calcium hydroxide (Ca(OH)_2_), provided hydroxide ions and directly regulated OH^−^ in the pore solution. The XCT image in Figure 12e shows less corrosion in zone 1, where a microcapsule is found on the upper left of the bar surface. In contrast, the region of zone 2 shows significant rusting on the bottom-right of the bar surface, where there is an absence of microcapsules.

Figure 7b,c show the corrosion process in the samples with the microcapsules. These samples had a lower volumetric loss throughout the experimental process compared to the control sample (Figure 10). The corrosion in the control sample developed more locally. In the samples with microcapsules, the corrosion was distributed in a relatively uniform manner on the reinforcing bars (Figure 7 and Figure 8). Thus, corrosion caused stress concentrations that led to cracks in the mortar matrix in the control sample. At the end of the experiment, after 1008 h of wet-dry cycles, Samples B and C had 0.675 mm^3^ and 0.823 mm^3^ of steel volumetric loss, respectively, but still no cracks could be found on them, while Sample A cracked with a volumetric loss of only 0.626 mm^3^ (Figure 10 and Table 6). These results suggest that the intelligent OH^−^-regulated microcapsule provided excellent protection against reinforcement corrosion.

When more CaO/EC microcapsules were added, better corrosion protection performance was theoretically expected, since more CaO/EC microcapsules contribute to [OH^−^] regulation. Similar results were reported for the simulated concrete pore solution [29]. However, the control sample (Sample A) showed obviously severe localized corrosion, while sample with 0.5% microcapsules (Sample B) showed a relatively uniform corrosion in the height direction. The loss development and the evident fluctuations of the sample with 3% microcapsules (Sample C) presented more pits, but had relatively less area loss of the steel bar, like a transition from uniform corrosion to nonuniform corrosion, indicating that adding more OH^−^-regulated microcapsules in the cementitious materials would be counterproductive. At the same time, Sample D had a higher volumetric loss compared to Samples B and C and the *R*_p_ results in Figure 11 showed a similar trend. It seems that a saturation of the inhibitive efficiency occurred with the incorporation of more CaO/EC microcapsules. More microcapsules could provide more adequate regulation of OH^−^ but also increase ionically-conducting paths up to a certain concentration. The growth trend in volumetric loss for Sample D after cracking is basically the same as that of control sample (Sample A), indicating that an outflow of OH^−^ may cause an inconspicuous effect on anti-corrosion of the reinforcing bar.

## 5. Conclusions

From the above analysis and discussion, the following conclusions can be made:

1. Intelligent OH^−^ regulation as a corrosion inhibitor or pH buffer in combination with pH-sensitive microcapsules was successfully designed to achieve and prevent chloride-induced corrosion in concrete reinforcing bars.
2. The X-ray computed tomography (XCT) method was used to trace and contrast the corrosion profiles of the concrete reinforcement bars with and without the microcapsules. The efficiency of the microcapsules was verified by XCT testing, which allowed visualization of the corrosion process (corrosion product, pitting, and cracking) and provided quantitative information.
3. The microcapsules showed highly effective corrosion protection due to intelligent OH^−^ regulation in the concrete material, which delayed corrosion initiation and cracking of the cover concrete.
4. An excessive addition of microcapsules may compromise the inhibitive efficiency of OH^−^ regulation.

## Figures and Tables

**Table 1:** Raw materials and ratios used for microcapsule fabrication.

Content	Ingredients	Mass (g)	Product
Core	Calcium hydroxide	57	Analytical reagent
	MCC	39	Chemically pure
	HPMC	2	Chemically pure
	Polysorbate 80	2	Chemically pure
Solvent	Deionized water	150	

**Table 2:** Preparatory materials for the capsule shell.

Content	Ingredients	Mass ratio	Product
Shell	Ethylcellulose	10	Chemically pure
Solvent	Methylbenzene	80	Analytical reagent
	Ethanol	20	Analytical reagent

**Table 3:** Microcapsule content in different samples.

Sample	Microcapsule	Dosage (wt.%)	W/C/S
A	–	0	2:5:5
B	CaO	0.5	2:5:5
C	CaO	3	2:5:5
D	CaO	5	2:5:5

**Table 4:** Elemental composition of the Q235 reinforcement.

	Fe	C	Mn	Si	P	S	Cr
Content (wt%)	97.64	0.195	1.56	0.57	0.023	0.004	0.008

**Table 5:** Mineral composition (wt%) of the cement.

SiO_2_	Al_2_O_3_	Fe_2_O_3_	CaO	MgO	C_3_S	C_2_S	C_3_A	C_4_AF
18.96	6.05	3.42	63.22	1.21	65.35	5.06	10.23	10.40

**Table 6:** Basic information of the XCT measurements.

Parameter	Value
X-ray source excitation voltage	70 kV
X-ray source excitation current	114 µA
Magnification	0.39×
Slice pixel number	1024 × 1024
Slice number	1000
Voxel size	15.0173 × 15.0173 × 15.0173 µm^3^

**Table 7:** Volume and volumetric loss of the reinforcing bar with different wet-dry cyclic times for the four samples.

Time *i* (h)	Sample A		Sample B		Sample C		Sample D	
	*V_i_*/mm^3^	∆*V*/mm^3^	*V_i_*/mm^3^	∆*V*/mm^3^	*V_i_*/mm^3^	∆*V*/mm^3^	*V_i_*/mm^3^	∆*V*/mm^3^
0	26.163	0.000	27.129	0.000	27.186	0.000	26.765	0.000
72	26.085	0.078	27.113	0.016	27.184	0.003	26.728	0.037
144	25.947	0.216	27.099	0.030	27.161	0.025	26.667	0.098
216	25.735	0.428	27.086	0.043	27.138	0.048	26.513	0.233
288	25.537	0.626	27.060	0.069	27.101	0.085	26.452	0.293
360	25.377	0.786	27.029	0.099	27.055	0.131	26.443	0.312
432	25.099	1.064	26.962	0.167	27.019	0.168	26.428	0.337
504	24.972	1.191	26.913	0.216	26.976	0.210	26.399	0.367
576	24.431	1.731	26.876	0.253	26.934	0.252	26.275	0.490
648	23.972	2.191	26.814	0.315	26.880	0.306	26.193	0.573
720			26.762	0.366	26.825	0.362	26.047	0.718
792			26.694	0.435	26.743	0.443	25.816	0.949
864			26.628	0.501	26.626	0.560	25.552	1.213
932			26.544	0.585	26.471	0.715	25.185	1.581
1008			26.454	0.675	26.364	0.823	24.598	2.167
